# Flexibility in female spatiotemporal behavioral tactics to counter infanticide risk during the mating season

**DOI:** 10.1186/s40462-025-00561-6

**Published:** 2025-05-19

**Authors:** Rick W. Heeres, Martin Leclerc, Shane Frank, Alexander Kopatz, Fanie Pelletier, Andreas Zedrosser

**Affiliations:** 1 Department of Natural Sciences and Environmental Health, University of South-Eastern Norway, Bø, Norway; 2 Département des Sciences Fondamentales and Centre d’étude de la Forêt, Université du Québec à Chicoutimi, Chicoutimi, Quebec Canada; 3 Mammals Research Section, Colorado Parks and Wildlife, Fort Collins, CO USA; 4 Norwegian Institute for Nature Research, Trondheim, Norway; 5 Département de Biologie, Université de Sherbrooke, Sherbrooke, Quebec Canada; 6 Institute of Wildlife Biology and Game Management, University of Natural Resources and Life Sciences, Vienna, Austria

**Keywords:** Conspecific associations, Encounter area, Home range, Solitary large carnivore, Proximity, Reproduction, Social behavior, Spatiotemporal movement, *Ursus arctos*, Sweden

## Abstract

**Background:**

Parental care is exclusively provided by females in most mammals, and mothers use several spatiotemporal behavioral tactics to minimize risks to offspring and to enhance fitness of both the mother and offspring. In species with infanticide and varying maternal care duration, dependent offspring remain vulnerable to male infanticide until separation from the mother. However, extending maternal care likely results in parent–offspring conflicts. We investigated the spatiotemporal behavioral tactics of lone female brown bears and mothers accompanied by offspring of varying ages in relation to infanticide risk and offspring separation during the mating season.

**Methods:**

We used data from 144 individuals (92 females and 52 males, 2003–2022) to characterize female spatiotemporal behavioral responses to males during the mating season by contrasting home range and encounter area sizes, proximity to males, and dyadic associations in relation to female reproductive status. We investigated the spatiotemporal behavioral responses of mothers from a socio-spatial perspective by connecting large-scale movement behavior (home range and overlap) and small-scale social behavior (proximity and associations) of adult females and males.

**Results:**

We found that females with dependent offspring of any age avoided males during the mating season. In comparison, lone females or mothers that lost or separated from their offspring during the mating season used larger areas and moved in closer proximity to males. The home range of mothers that remained with their offspring still largely overlapped with male home ranges, however, they did not associate (< 100 m) with males. Additionally, mothers with yearlings had similar sized home ranges as solitary females, but larger home ranges in comparison to mothers with cubs-of-the-year. This suggests that mothers with yearlings are more conspicuous on the landscape which may result in a higher detectability by males.

**Conclusion:**

Our results suggest that mothers with offspring of any age perceive adult males as potential source of infanticide and use spatiotemporal avoidance tactics. Generally, family groups had high home range overlap with adult males, but family groups that remained together throughout the mating season did not associate with any adult male. Mothers with yearlings used larger areas in comparison to mothers with cubs, potentially indicating their increasing energetic needs. The use of spatiotemporal behavioral tactics to avoid infanticide by females with dependent offspring irrespective of age likely disrupts movement, mating, and social dynamics and on the long-term potentially increases the risk of infanticide to older offspring.

**Supplementary Information:**

The online version contains supplementary material available at 10.1186/s40462-025-00561-6.

## Background

Mating systems include various spatiotemporal behavioral tactics to increase mating opportunities and reproductive success [1, 2]. Tactics can vary inter- and intra-individually within and between years [3] due to an individual’s sex, age, or body/weaponry size [4]. In addition, the spatiotemporal distribution of resources at the population level, such as food, mates [5] and the operational sex ratio [6], can influence the choice of mating tactics. In the long-term, anthropogenic (e.g., hunting), environmental (e.g., climate), and social selection of behavioral tactics can influence population level processes and may reduce the mean fitness in a population [7, 8]. For example, the selective removal of males can lead to skewed age structures resulting in lower fecundity, delayed parturition date, and reduced recruitment due to female hesitation to mate with young males or insufficient number of males [9].

In mammals, parental care is always provided by females [10, 11] with biparental care only found in few species [12]. Female maternal care tactics include behaviors that reduce predation risks to offspring [13, 14], thereby enhancing future fitness of both mother and offspring [15, 16]. Maternal care duration (defined as time from birth to separation) is influenced by factors such as duration of lactation and weaning period, because some species have an extended weaning period after the cessation of lactation [10, 15, 17]. Across mammal species, there is large variation in the duration of maternal care, across and within populations, spanning from days up to multiple years [18, 19]. Extending maternal care allows females to allocate more resources to the fitness of current offspring at the expense of future reproduction [20, 21]. Thus, longer maternal care could result in parent–offspring conflicts [22, 23] due to increasing nutritional demand and/or competition with the growing offspring for food resources and cause mothers to adapt through changes in behaviors such as space use (e.g., pre- and postpartum daily movement or home range sizes; [24, 25]).

Premature loss of offspring for any reason releases the female from maternal care and results in changes in spatiotemporal behavioral tactics, e.g., from avoiding to associating with conspecifics [26]. Infanticide is a common cause for the loss of dependent offspring [27, 28], and males may kill offspring that they have not sired to gain reproductive access to the mother, i.e., sexually selected infanticide (SSI; [29]). This male mating tactic capitalizes on the ability of females to rapidly enter estrus after offspring loss [30, 31]. For example, abrupt termination of maternal care due to SSI triggers the initiation of the next reproduction in *Felid* spp. or *Ursid* spp. within days or weeks [18, 31, 32]. Females have developed counterstrategies against SSI, such as mating with dominant males, defending offspring, or mating with multiple males creating paternity confusion [29, 33, 34]. Females with dependent offspring might also spatiotemporally avoid males during the mating season [14, 35, 36]. In species with several years of maternal care, SSI can involve dependent offspring of any age, as long as the offspring receives care [37, 38]. However, as offspring mature, females might alter their spatiotemporal behavioral tactics countering SSI risk as older offspring may be able to evade SSI and/or survive on their own in case of separation after an infanticidal attack.

In brown bears (*Ursus arctos*), SSI is a common male mating strategy [39–41]. In hunted populations, the occurrence of SSI is higher due to disrupted social structures, with more incoming males in response to local vacancies [41, 42]. SSI events occur during the mating season [31, 43], which lasts from May until July and coincided with the most frequent male–female associations [44]. Females with cubs-of-the-year (0.5 years old; hereafter cubs) adjust their spatiotemporal behavioral tactics by spatially segregating from males during the mating season (male avoidance hypothesis; [45, 46]). Maternal care duration in brown bears lasts 1.5–2.5 years in Europe [47], up to 3.5 years in North-America [48, 49], and even 4.5 years in Middle-Eastern/Asian populations [50]. The survival of dependent offspring varies among age classes [50, 51]. SSI is the dominant cause of death for cubs [39, 41], but may also occur in older offspring [52]. Therefore, SSI risk might be one of the drivers of female spatiotemporal behavior when accompanied by offspring. Besides SSI, family breakup usually occurs during the early stages of the mating season [45]. Thus, investigations during the mating season are essential for understanding female spatiotemporal behavioral tactics in relation to age, and events such as loss of offspring and the breakup of family groups.

**Fig. 1:**
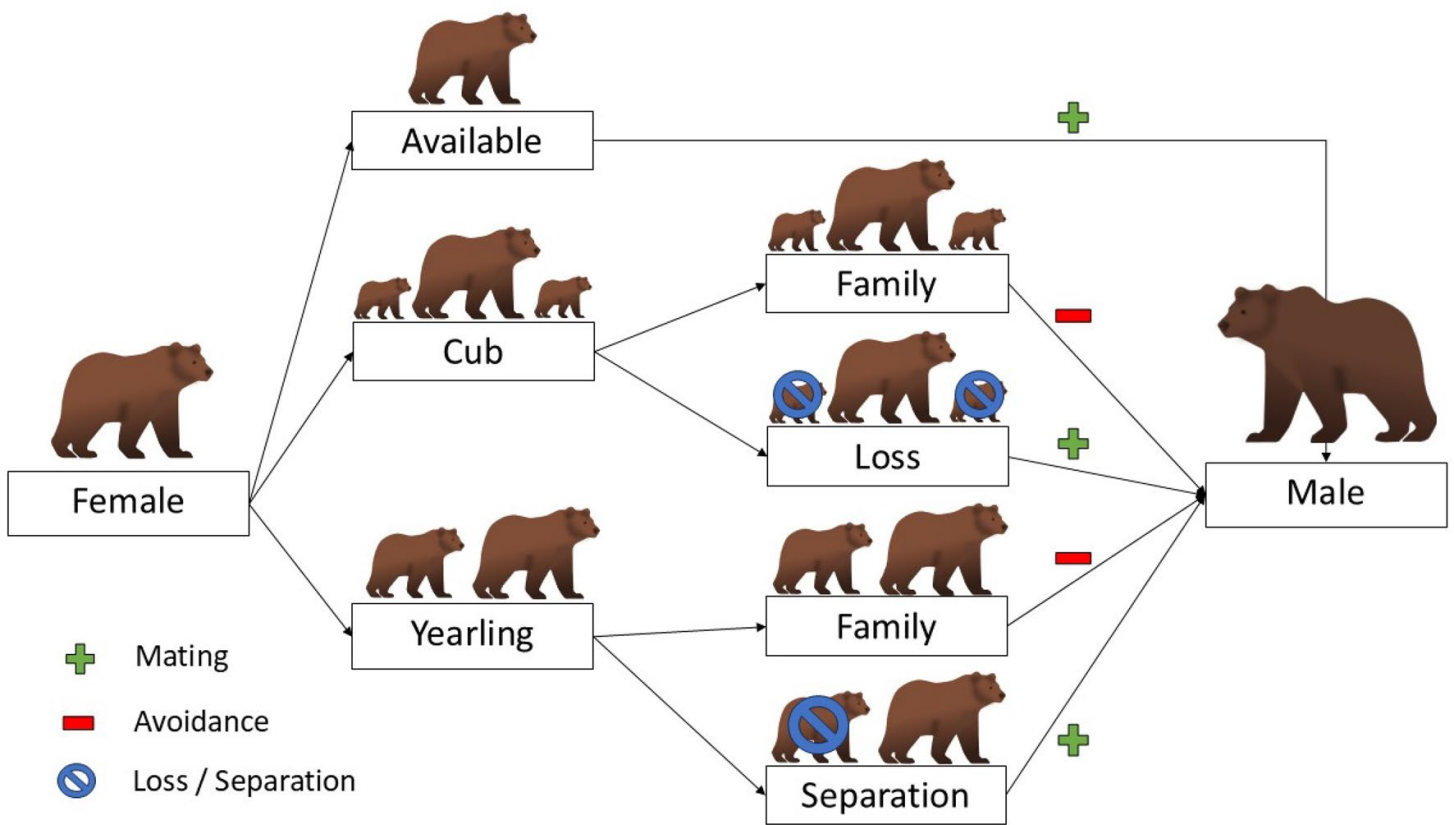
Flowchart of all possible combinations of a female brown bear’s reproductive status and the occurrence of a separation event (offspring loss, sexually selected infanticide or family break-up), and the respective predictions per classification. Sample sizes per group were: 22 cub family, 20 cub loss, 37 yearling family, 39 yearling separation, and 151 available females. A plus (+) symbolizes a behavioral tactic where a female has a larger home-range and encounter area, associates with males, and remains near males during the mating season. A minus (−) refers to a behavioral tactic where females avoid males, have a smaller home-range and encounter area, and remain distant from males during the mating season

Previous studies on Scandinavian brown bears showed that females with cubs increased their movement speed after family separation [31] and that females with cubs and yearlings spatially segregate as they utilize different habitats compared to adult males [53], [54]. Here, we build on these previous studies and investigate if females with dependent offspring of any age avoid adult males by focusing on both movement and social behavior (i.e., socio-spatial interface; [55]). We use GPS data of brown bears to compare spatiotemporal behavioral tactics (hereafter; behavioral tactics) of females accompanied by yearlings (1.5 years) to tactics by solitary females and females with cubs during the mating season. We investigate the occurrence and timing of female-male dyadic associations, contrast home range size and proportion of female-male home range overlap, and the proximity of females to males in relation to a females’ reproductive status. The combination of these methods allows the comparison of female behavioral tactics at different socio-spatial scales, ranging from large scale movement patterns (i.e., home range and overlap) to small scale social patterns (i.e., proximity and associations). Solitary females aim to maximize their mating opportunities by increasing their home range size during the mating season [56]. In comparison, females with cubs spatiotemporally avoid males during the mating season [46], but rapidly switch their behavior after loss of their cubs during the mating season to maximize their reproductive opportunities [31]. However, the behavioral tactics of females with older dependent offspring remain largely unknown [54]. Female brown bears with yearlings exhibit two maternal care tactics in the study population [57]. They either separate (1.5 years; short-term strategy) or keep the yearlings for an additional year (2.5 years; long-term strategy). When a female retains her yearlings, they likely remain vulnerable to SSI [52] which should be reflected in the females’ spatiotemporal behavior towards males. Therefore, we predict that (1) the behavior of females retaining their yearlings is similar to the that of females with cubs (male avoidance hypothesis), and (2) the behavior of females separating from their yearlings due to SSI or family break up is similar to that of solitary females (maximized mating hypothesis). Females following the “male avoidance hypothesis” should exhibit limited associations with males, have smaller home ranges and lower overlap with males, and remain far from males during the mating season. In comparison, females following the “maximized mating hypothesis” should exhibit frequent associations with males, have larger home ranges and higher overlap with males, and remain closer to males during the mating season (Fig. 1 & Appendix S6: Fig. S6).

## Methods

### Study system

The study area covers ~13,000 km^2^ of managed coniferous forests, bogs, and lakes in south-central Sweden (~61°N, 15°E; [58]). The bear density in the area is ~23 individuals per 1000 km^2^ [59]. Swedish brown bears are legally hunted from 21 August to 15 October or until quotas are filled, and ~10% of the total population are harvested annually [59]. Hunting has profound effects on the movement behavior, social structure, and mating system of our study population [42, 60, 61].

Brown bears are non-territorial and solitary-living carnivores with a polygamous mating system [62], and the mating season lasts from May 15 to July 21 in Sweden [44]. Social associations between solitary individuals are mainly driven by reproduction and peak during the mating season, though associations also occur during the remainder of the active period [44]. Maternal care lasts either 1.5 or 2.5 years in Sweden [47], likely depending on maternal-offspring condition, litter size and anthropogenic factors [57].

### Brown bear captures and monitoring

Individuals were captured annually as part of a long-term monitoring project after den emergence in spring (i.e., late April, early May). Bears were darted from the helicopter and equipped with GPS-collars (GPS Plus; Vectronic Aerospace) programmed to relocate the individual every hour. See Graesli et al. [63] for more details on capture and handling. All aspects of animal capture and handling were approved under ethical permits provided by the Uppsala Ethical Committee on Animal Experiments (Dnr 5.8.18-03376/2020). The capture permit was provided by the Swedish Environmental Protection Agency (NV-01278-22).

### Female reproductive status and offspring separation

We selected females ≥ 4 years, as the majority of primiparity occurs at age 4 or older [62, 64]. However, males can reproduce as early as 3 years old in Scandinavia. Age (e.g., year of birth) was known for most bears because they were captured as part of a family group. For bears not followed from birth, a premolar tooth was extracted for age determination [65]. Female reproductive status was determined at capture or via visual observations several times per year via helicopter [57]. We classified female reproductive status during a mating season as: *available,* when the female was solitary and observed without dependent offspring after den emergence; with *cubs*, when the female was observed with dependent cubs-of-the-year after den emergence; or with *yearlings* if the individual was observed with dependent one-year old offspring after den emergence. Females accompanied by two-year-old offspring were also classified as *available* as they separate shortly after leaving the den. Frequent monitoring from a helicopter allowed us to detect if and when a female became available, however, we were not able to determine whether this change in status was caused by offspring loss (via SSI or starvation) or family break-up. We combined a female’s reproductive status and the occurrence of a family separation event to produce the following female classifications; available, cub family, cub loss, yearling family, and yearling separation (Fig. 1). We differentiated between “cub loss” and “yearling separation” as cubs in comparison to older offspring would not be able to survive if separated from their mother during the mating season.

### Home range

We calculated individual home ranges for adult females (*n* = 92; ≥ 4 years; 269 bear-years) and males (*n* = 43; ≥ 3 years; 102 bear-years) with data available for the mating season. Further, we verified that all variograms reached a plateau to obtain robust estimates and range residency (Appendix S1: Fig. S1; [66]). Then, we calculated 95% kernel density estimates (KDE) for all males and 229 female bear-years with the *ctmm* package [67], as we were interested in the area used during the mating season and not the individuals’ future or potential range [68]. We used one-way ANOVA to evaluate differences in average home range size estimates among female groups [69]. We log-transformed home range sizes to obtain normality and checked for homogeneity of variance using Levene’s Test [69]. We applied Tukey’s “Honest Significant Differences” method (HSD; 95% confidence level) to significant ANOVA results (*p* < 0.05) to investigate the pair-wise comparisons of means among the female classifications. This method takes into account errors related to multiple comparison among groups [70]. We used R 4.4.1 [71] for all analyses.

### Encounter area overlap

Based on the KDE estimates, we calculated the conditional location distribution of encounters (CDE; hereafter encounter areas) between females and males with home range estimates [72]. An encounter area describes the home range overlap distribution of two individuals accounting for their movement behavior. We then used the “overlap” function in the *ctmm* package to determine the proportion of each individual’s home range area that fell within the encounter area. We removed all estimates between individuals that did not share an encounter area or the area was too small to be informative (estimate < 1 km^2^; 170 female bear-years). Overlap estimates were analyzed with a Kruskal–Wallis (KW) test to assess significant differences between the proportional overlap estimates of the female classifications [73]. We applied a Pairwise Wilcoxon Rank Sum test using a “holm” multiple comparison correction [74] on significant (*p* < 0.05) KW test results to investigate the pair-wise comparisons of overlap estimates among female classifications.

### Proximity to males

To determine proximity events between females and males, we calculated all pairwise distances between females and males utilizing hourly GPS relocations during the mating season (2003–2022) with the *spatsoc* package [75]. The females included in the proximity analysis overlapped with at least one male (within 100% Minimum Convex Polygon (MCP),186 female bear-years). We used a threshold of 5000 m, as this is the average daily total distance travelled by a female [76] at a mean movement rate of 382 m per hour [44]. The distance was only estimated if both the female and male had a GPS-fix at the same hour interval (tolerance of ± 3 min).

We fit a Hierarchical Generalized Additive Mixed Model (HGAM; [77]) using the *mgcv* package [78] with a Tweedie distribution (log-link) and Restricted Maximum Likelihood (REML) variance estimator [79] to the hourly distance between females and males and used female classifications as a predictor. We expected the effect of day of the year (*doy*) to vary among female classifications (e.g., different predictions in terms of distance to males), therefore we added a factor-smoother interaction between the *doy* and the five female classifications [80]. Additionally, female classification was added as a random effect, because group-specific intercepts are not incorporated into factor-smoothers [77]. In addition, we added the random effects ‘bear-year’ (unique female identity) to consider individual differences, and ‘encounter ID’ to control for pseudo-replication of hourly distances for the same dyad during the ongoing mating season. We used the *DHARMa* [81] and *mgcViz* [82] packages to check the HGAM model fit.

### Individual associations and timing

Similar to the proximity events (186 female bear-years), we calculated all pairwise distances between individual hourly GPS relocations to determine dyadic associations. We defined a dyadic association as two or more individuals within a Euclidean distance < 100 m of each other with a 3-min tolerance to avoid underestimation of the number of associations due to the relatively coarse fix rate. Additionally, a sensitivity analyses by Heeres et al. [44] suggests that 100 m is an adequate dyadic distance threshold to capture representative social structures and networks in this population. The data were used to investigate the occurrence and timing of female-male associations during the mating season and differences among female classifications.

## Results

### Home range

**Fig. 2:**
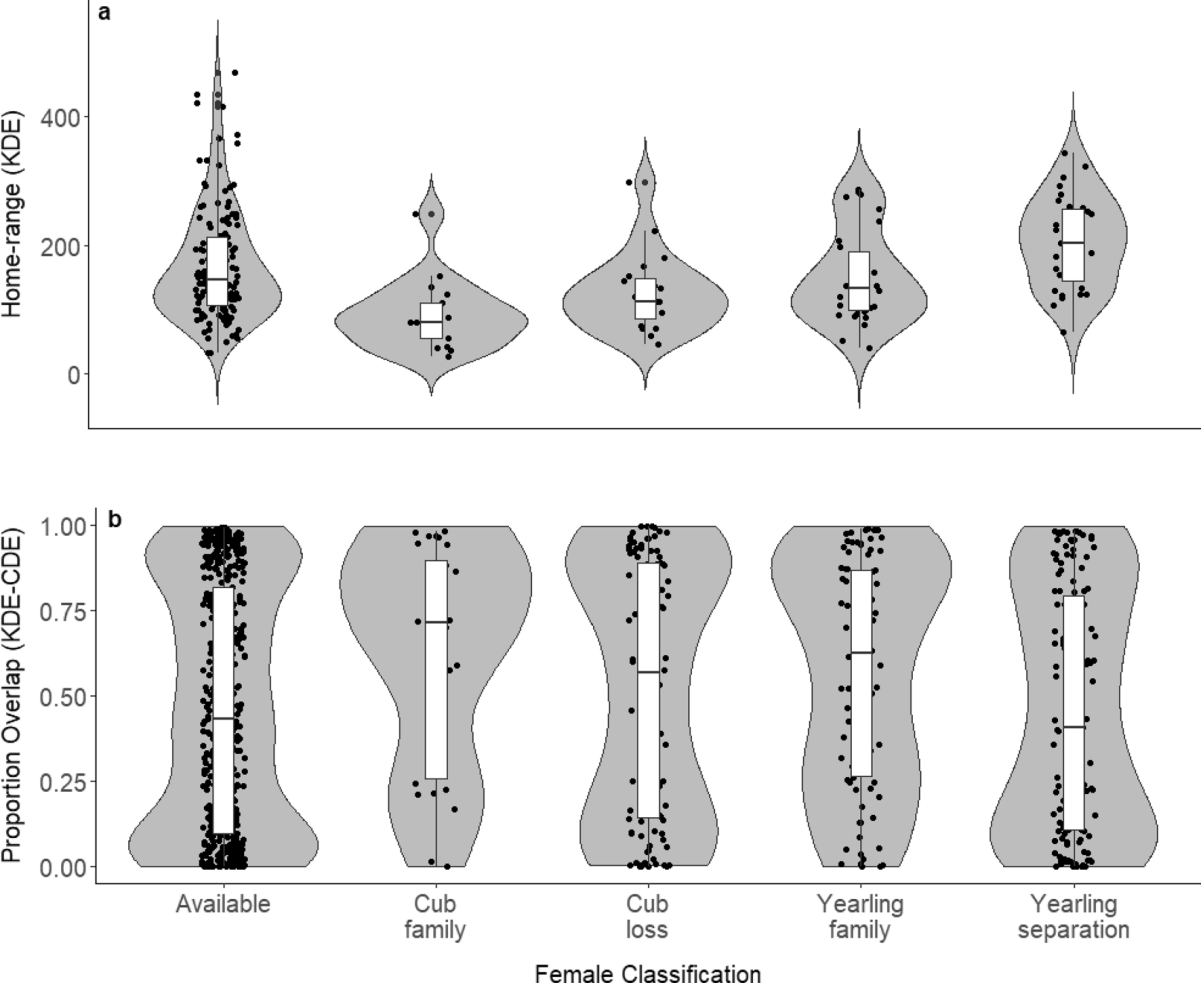
**a** Violin plot of the 95% kernel density estimates (KDE) estimates (km^2^) for female brown bears (229 bear-years with GPS locations) and **b** Violin plot of overlap estimates (individual overlap of 95% KDE and CDE) between females and males in km^2^ for females (170 bear-years; *n* = 822). We separated the estimates per female classification (incl. reproductive status and occurrence of separation event). All data were collected during the mating season in south-central Sweden between 2003 and 2022

We observed significant differences in home range size of females (229 bear-years) during the mating season based on their classification (ANOVA, *p* < 0.001, Fig. 2a & Appendix S2: Table S2.1). “Available” females (mean: 169 ± 87 km^2^) had significantly larger home ranges compared to “cub family” (92 ± 54 km^2^; Tukey HSD: *β* = 0.53, *p* < 0.001; Appendix S2: Table S2.2). Moreover, “cub family” had significantly smaller home ranges compared to “yearling family” (151 ± 72 km^2^; *β* = 1.69, *p* = 0.005) and “yearling separation” (205 ± 70 km^2^; *β* = 2.41, *p* < 0.001). “Cub loss” (126 ± 61 km^2^; *β* = 1.68, *p* = 0.003) and “yearling family” (*β* = 1.41, *p* = 0.047) had smaller home ranges than “yearling separation”. Lastly, “available” females had similar sized home ranges as females with yearlings (*p* = 0.865).

### Encounter area and overlap

The overlap analysis (Fig. 2b & Appendix S3: Table S3.1) indicated significant differences among female classifications (Kruskal–Wallis: *p* = 0.016; 170 bear-years, *n* = 822). However, we found no significant differences in overlap with males between the female classifications (Pairwise Wilcoxon Rank Sum test with Holm correction; *p* > 0.16 for all group comparisons; Appendix S4: Table S3.2). We also found no significant differences between encounter area size during the mating season among the different female classifications (ANOVA: *p* = 0.173; *n* = 822, Appendix S3: Fig. S3).

### Proximity females–males

**Fig. 3:**
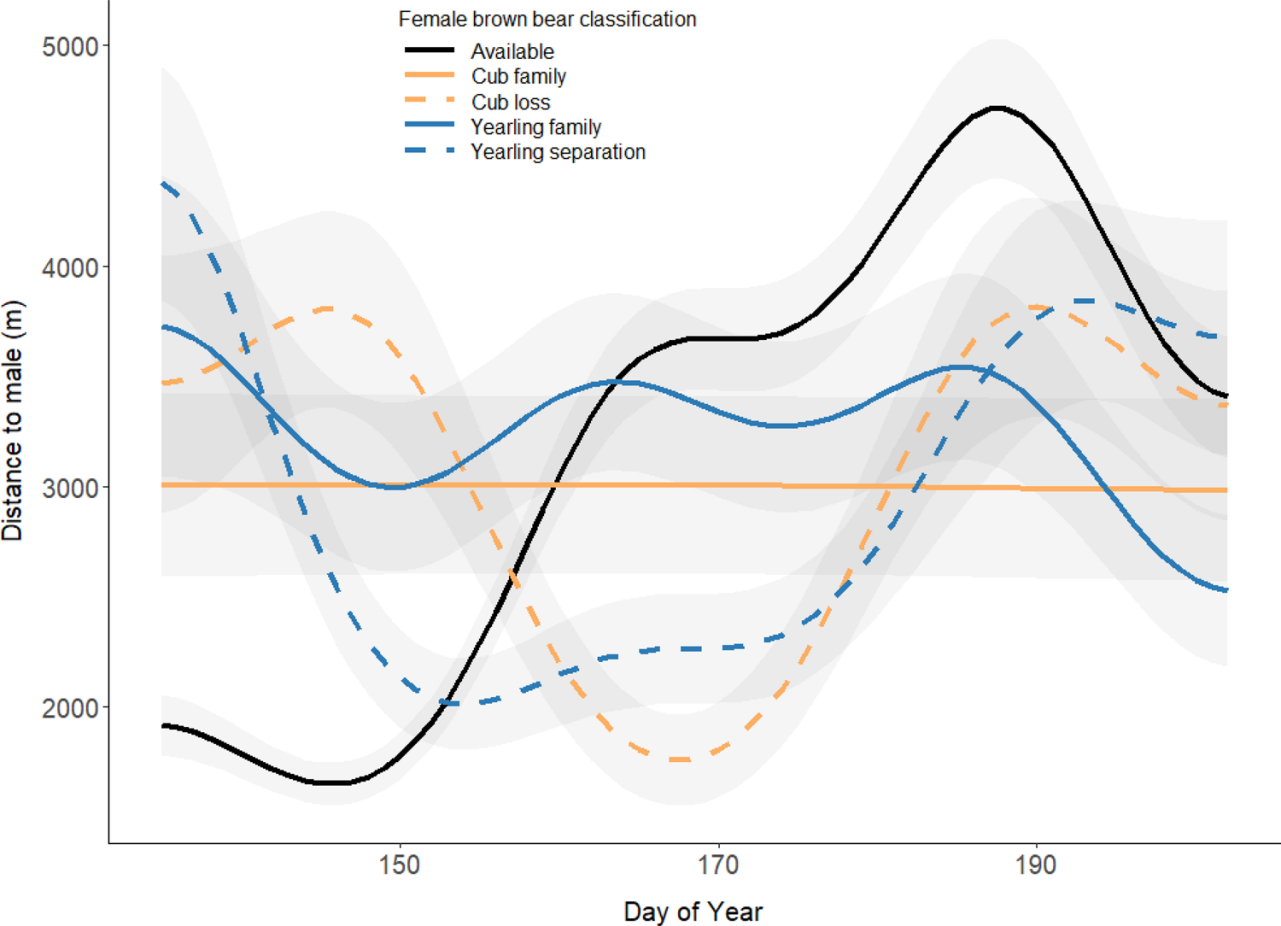
Hourly distances from female brown bears to males (*n* = 32,245; 142 bear-years) during the mating season (distance threshold = 5000 m) in relation to the day of the year, reproductive status and occurrence of separation [i.e., offspring loss/sexually selected infanticide (SSI) or family break-up]. The data was collected in south-central Sweden between 2003 and 2022. The *x*-axis encompasses the entire mating season (Julian date 135–202; May 15–July 21). The shaded area around the lines correspond to the 95% confidence intervals per female classification

We found different patterns in the hourly female-male distances (*n* = 32,245; *N* = 142 bear-years) based on a female’s reproductive status, day of the year, and the occurrence of an offspring separation event (Appendix S4: Table S4.1 & S4.2). We found the following patterns: (1) “available” females begin close to but gradually increase their distance to males during the mating season; (2) females that lose or separate from offspring begin far from males, move closer to males after the first weeks of the mating season, and then gradually increase their distance to males towards the end of the mating season; (3) females accompanied by offspring of any age remain far from males during the entire mating season (Fig. 3 & Appendix S4: Table S4.1). We also found that the daily travel distance differed considerable depending on the female's classification (Appendix S7: Fig. S7, Table S7.1 & Table S7.2). Females that lost or separated from their dependent offspring, both females with yearlings and two-year-olds, almost doubled their daily travel distance compared to females that remained with their offspring.

### Timing of dyadic associations

**Fig. 4:**
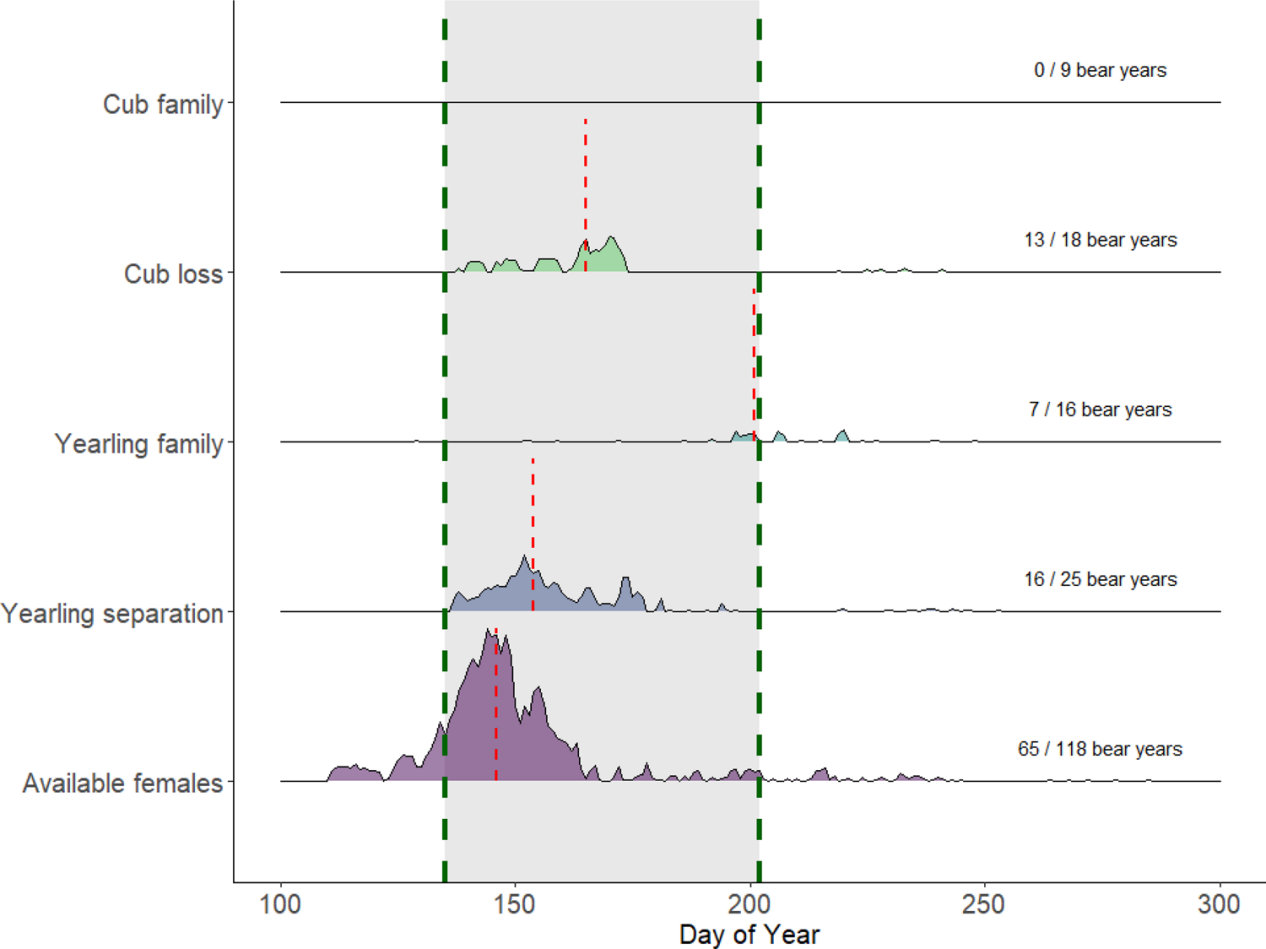
Frequency of female-male brown bear associations per day of the year (day 100 corresponds to April 10) per female classification (incl. reproductive status and occurrence of family separation or loss event). The data were collected during the mating season in south-central Sweden between 2003 and 2022. The maximum number of daily associations for the “available”, “yearling separation”, “yearling family”, and “cub loss” groups are 259, 96, 19 and 61 respectively. The mating season lasts from day 135 to day 202 (shaded area and green vertical dotted lines). The median date of associations per group is indicated by the red vertical dotted lines. The sample size for which we identified overlap with a male (186 bear-years) and associations (excl. “cub family”) is stated per group (101 bear-years)

We identified 8223 dyadic associations (*n* = 103 bear-years) between females and males (Appendix S5: Table S5.1). We found that females in family groups had almost no associations with males during the mating season (Fig. 4); i.e., no associations between males and females with cubs, and only sporadic associations (mean = 9/female, *n* = 65; Appendix S5: Table S5.1) for females with yearlings which predominantly occurred during the last five days of the mating season. In comparison, females that either lost or successfully separated from their offspring had 795–1600 associations (mean loss = 61/female; mean separation = 100/female) with males during the mating season. Lastly, “available” females had 5467 associations in total (mean = 84/female). The timing of female-male associations during the mating period was different among the female classifications (Fig. 4; Appendix S5: Table S5.1; Appendix S8: Fig. S8). Females with cubs or yearlings that experienced family separation or loss associated with males later during the mating season compared to available females.

## Discussion

Our objective was to characterize spatiotemporal behavioral tactics of females accompanied by offspring of varying age in relation to males during the mating season. We build on previous work contrasting habitat use of females with offspring and males [53, 54] and changes in female movement behavior after the loss of cubs [31], and investigated the behavioral tactics of females during the mating season. We connected both large-scale movement- and small-scale social behavior and found two distinct female tactics, i.e., (1) mothers in family groups had infrequent or no associations with males, smaller home ranges, and stayed farther away from males; in comparison, (2) lone females had multiple associations with males, larger home ranges, and were generally closer to males during the mating season. Upon losing offspring (e.g., either due to loss or separation), both females with cubs and females with yearlings switched from the first to the second tactic during the mating season. These females likely switch their behavioral tactic to maximize mating opportunities by having larger home ranges and associating with males, similar to “available” females (support *prediction 2*). Females retaining dependent offspring, irrespective of age, avoid males both spatially and temporally during the mating season (support *prediction 1*). However, the home range overlap estimates between females and males were high for all female classes, also for the ones with dependent offspring.

The probability that females retain their yearlings for an additional year has increased significantly in our study area over time [57]. In Sweden, family groups are protected from hunting [83], which likely promotes the long-term maternal care tactic (2.5 years) through increased protective status of females and their offspring during the hunt for an additional year [57]. This has resulted in a higher proportion of family groups with yearlings [84]. Therefore, males might have limited mating options and may opt to force separation or even kill yearlings [43, 52]. For males, females with offspring likely represent a more costly mating opportunity in terms of energy used to try to kill the offspring and/or to cope with female aggression [29]. Therefore, such females may potentially be avoided or ignored until all other available females have been mated [30, 36, 45]. We observed a delay in timing of associations with males between “available” females, females that separated from yearlings, and females that lost cubs. Additionally, females with dependent offspring often had high spatial overlap with males, suggesting that these females are either ignored or not detected by males. These females might be ignored when moving in the home range of a male they had mated with the previous year (paternal confusion; [29]). However, as SSI is a common male mating strategy [39], any male that had not mated with the female the previous year should approach her when she is accompanied by offspring and either attempt to kill the offspring or force a family breakup.

In ungulates, antipredator tactics shape the movement patterns and therefore the resource exploitation of females during maternal care [85]. In addition, a females’ home range generally increases with increasing offspring age, in addition to moving towards higher quality habitat to facilitate increasing nutritional demands [24]. Our study shows that the avoidance of a predator (i.e., here male bears) by female brown bears with cubs is also apparent in females with yearlings. Spatiotemporal segregation is a known tactic of female brown bears with dependent offspring during the mating season [46, 54]. In addition, females often use areas that are generally avoided by males during the mating season (“human shield” hypothesis; [53]). However, the spatiotemporal segregation likely forces family groups into poorer resource areas [46, 86, 87]. The home range sizes were larger for females with yearlings in comparison to those with cubs. This increase in space use could potentially indicate a parent–offspring conflict which urges females, especially those with yearlings, to make riskier decisions in facilitating her and the offspring’s energetic needs [25, 88]. Females with yearlings also had home ranges sizes similar to solitary females, which indicates that the resource needs increase for families with yearlings [62]. In addition, Van de Walle et al. [57] showed that larger yearling litter sizes are associated with extended maternal care, which again supports the increased resource needs for mothers keeping yearlings for an additional year. Additionally, increased space use is accompanied by increased energetic demands for the whole family, and must be compensated for [89]. Alternatively, females with yearling may be less constrained in their movements compared to females with cubs which may potentially explain the increase in space use by female with yearling.

The duration of parental care affects the tradeoff between offspring quantity (maximizing the number of offspring produced) versus offspring quality, i.e., fewer but larger offspring with potentially high lifetime fitness; [11, 17, 90, 91]). The probability of continuing maternal care in our population was higher for females with larger litters (2–3 cubs; [57]). These females have to allocate more energy to their current offspring which may reduce their future reproduction especially because they reduce their reproductive opportunities [18, 92, 93]. The extended maternal care period might provide the offspring with additional survival and fitness benefits such as enhanced social skills [61, 94] or equip the offspring with crucial skills taught by their mother [20, 95]. However, if an increasing number of females extend their maternal care, the infanticide pressure on yearlings might increase and thereby reduce any potential fitness benefits of prolonged maternal care.

Unraveling the behavioral adaptability of females in response to risk of infanticide during the mating season is crucial for advancing our knowledge regarding fitness, reproduction, and evolutionary dynamics [4, 55, 96]. Our study supports the contention that wildlife management practices can have cascading effects on movement, reproduction, as well as the social composition of populations [97, 98]. The increase in the length of maternal care in Sweden during the last decade may partially be related to hunting, as females with family groups are protected from hunting which may have resulted in increased survival and a selective advantage of females with prolonged maternal care [57, 62]. In 2022-2024, Swedens substantially increased the annual harvest rate from 10 to almost 25% in order to reduce the population size and density [99]. It remains to be seen how this strong increase in the harvest rate will affect movement- and social behavior, maternal care tactics, and ultimately population dynamics.

## Supplementary Information

Additional file1 (DOCX 4063 kb)

## Data Availability

Data that supports the findings is available in the University of South-Eastern Norway Research Data Archive, which can be found here: https://doi.org/10.23642/usn.24949185.v2
