# Supplementary material for: Flexibility in female spatiotemporal behavioral tactics to counter infanticide risk during the mating season
Source: Mov Ecol. 2025 May 19;13:35. doi: 10.1186/s40462-025-00561-6 (PMC12090450; doi:10.1186/s40462-025-00561-6)
Supplement: Supplementary file 1 — Additional file1 (DOCX 4063 kb) [file 40462_2025_561_MOESM1_ESM.docx]

**Appendix S1. Example of a home-range variogram**


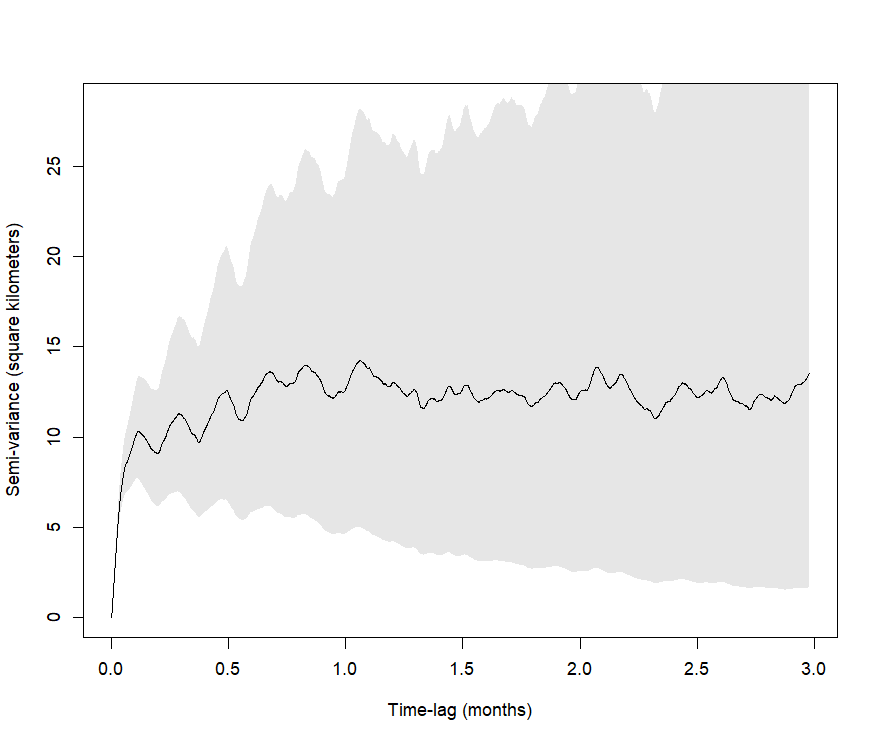


Figure S1: Variogram for individual W0229 (2010), showing the variance regarding the size of the kernel density estimates (KDE) over time.

**Appendix S2. Home-range analysis**

Table S2.1: Summary statistics for the number of female brown bears (bear-years = 229) per reproductive status and occurrence of separation event (infanticide or family break-up) used for the calculations of the 95% KDE areas (km^2^) between 2003-2022 during the mating season in south-central Sweden.

| Reproductive status | Separation | Bear-years | Mean km^2^ (± SD) |
| --- | --- | --- | --- |
| Available | - | 132 | 169 ± 87 |
| Cub | Family | 17 | 92 ± 54 |
|  | Loss | 19 | 126 ± 61 |
| Yearling | Family | 30 | 151 ± 72 |
|  | Separation | 31 | 205 ± 70 |

Table S2.2: Output of the Tukey’s *Post-Hoc* Comparison test using the KDE ANOVA results based on the KDE 95% log-transformed estimates for female brown bears between 2003-2022 during the mating season in south-central Sweden (bear-years = 229). Significant comparisons are shown in **bold** (< 0.05 threshold). The comparisons are between all female classifications (incl. reproductive status and the occurrence of separation event such as offspring loss/infanticide or family break-up).

| KDE ANOVA (Tukey’s test) | | | | |
| --- | --- | --- | --- | --- |
| *Group 1* | *Group 2* | *Estimates* | *CI (95%)* | *p* |
| Cub family | Available | 0.53 | 0.38 – 0.76 | **<0.001** |
| Cub loss | Available | 0.77 | 0.55 – 1.07 | **0.197** |
| Yearling family | Available | 0.91 | 0.69 – 1.20 | **0.865** |
| Yearling separation | Available | 1.29 | 0.98 – 1.69 | 0.081 |
| Cub family | Cub loss | 1.44 | 0.91 – 2.27 | 0.192 |
| Yearling family | Cub loss | 1.69 | 1.12 – 2.57 | **0.005** |
| Yearling separation | Cub loss | 2.41 | 1.60 – 3.64 | **<0.001** |
| Yearling family | Cub family | 1.18 | 0.79 – 1.76 | 0.787 |
| Yearling separation | Cub loss | 1.68 | 1.13 – 2.50 | **0.004** |
| Yearling separation | Yearling no separation | 1.42 | 1.00 – 2.02 | **0.047** |

**Appendix S3. Encounter area overlap analysis**

Table S3.1: Overview of number of female brown bears (170-bear years), number of calculated encounter areas (*n* = 822), and mean area size (km^2^) per female reproductive status and the occurrence of separation event (loss or family break-up) regarding the calculations of the encounter areas between 2003-2022 in south-central Sweden.

| Reproductive status | Separation | Bear-years | Samples (n) | Mean km^2^  (±SD) |
| --- | --- | --- | --- | --- |
| Available | - | 98 | 477 | 69 ± 59 |
| Cub | Family | 9 | 26 | 48 ± 29 |
|  | Loss | 18 | 102 | 53 ± 38 |
| Yearling | Family | 19 | 81 | 60 ± 39 |
|  | Separation | 26 | 136 | 76 ± 60 |

Table S3.2: Output of the Pairwise Wilcoxon Rank Sum test using the proportional overlap log-transformed estimates (calculated using individual 95% KDE and CDE) of female brown bears (170 bear-years and *n* = 822) between 2003-2022 during the mating season in south-central Sweden. The comparisons are between all female classifications (incl. reproductive status and the occurrence of separation event such as offspring loss/infanticide or family break-up).

| *Group 1* | *Group 2* | *p* |
| --- | --- | --- |
| Available | Cub family | 0.180 |
| Available | Cub loss | 0.880 |
| Available | Yearling family | 0.160 |
| Available | Yearling separation | 1.000 |
| Cub family | Cub loss | 0.920 |
| Cub family | Yearling family | 1.000 |
| Cub family | Yearling separation | 0.180 |
| Cub loss | Yearling family | 1.000 |
| Cub loss | Yearling separation | 0.920 |
| Yearling family | Yearling separation | 0.180 |


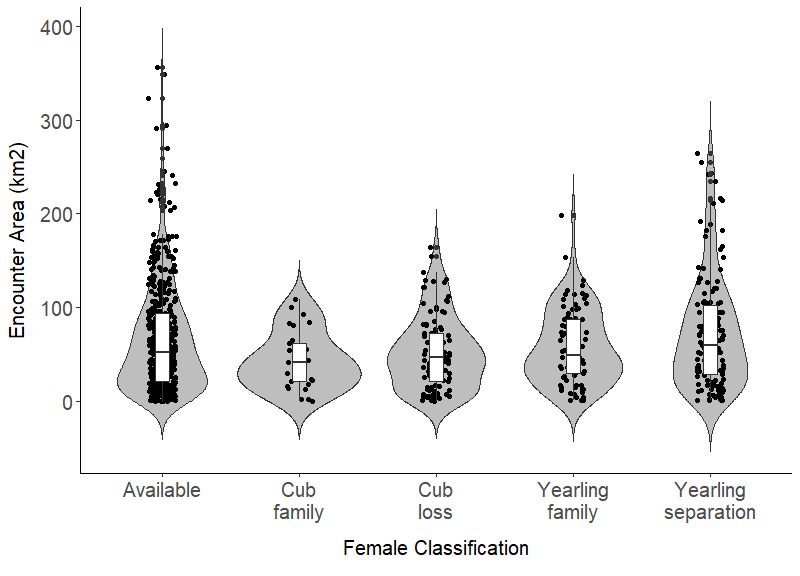


Figure S3: Boxplot of the 95% CDE estimates (km^2^) for all female brown bears (170 bear-years; *n* = 822) and separated per female classification (reproductive status and occurrence of separation). The data was collected during the mating season in south-central Sweden between 2003-2022.

**Appendix S4. GAMM distance analysis: sample size and results**

Table S4.1: Overview of number of unique female brown bears (142 / 186 bear-years) and total samples (*n* = 32,245) per reproductive status and occurrence of separation event between 2003-2022 during the mating season in south-central Sweden. The mean daily distance to males (distance threshold = 5000m) is also indicated, including the standard deviation.

| Reproductive status | Separation | Bear-years | Total samples | Mean distance (m ± SD) |
| --- | --- | --- | --- | --- |
| Available | - | 82 (118) | 18,648 | 2234 ± 1753 |
| Cub | Family | 8 (9) | 535 | 3198 ± 1279 |
|  | Loss | 17 (18) | 3,919 | 2376 ± 1776 |
| Yearling | Family | 14 (16) | 3,735 | 3200 ± 1314 |
|  | Separation | 21 (25) | 5,408 | 2118 ± 1806 |

Table S4.2: Smooth term estimates (log-transformed) from the proximity GAMM analysis (reference = available females) regarding the average distance from female brown bears to males (distance threshold = 5000m) per reproductive status and occurrence of infanticide or family break-up event during the mating season per female classification (142 bear-years, n = 32,245 hourly distances).

| **Average daily distance females – males (log-transformed)** | | |
| --- | --- | --- |
| *Smooth term^a^* | *edf* | *p* |
| Available * doy | 4.93 | **<0.001** |
| Cub family * doy | 0.07 | 0.340 |
| Cub SSI * doy | 4.75 | **<0.001** |
| Yearling family * doy | 4.25 | **0.025** |
| Yearling separation * doy | 4.77 | **<0.001** |

^a^ We used the day of the year (*doy*) variable as a factor-smoother interaction in the GAMM.

**Appendix S5. Summary statistics for the dyadic associations**

Table S5.1: Summary statistics of the number of female brown bears (103 / 186 bear-years), dyadic associations (*n* = 8223) and average number of associations per female classification (reproductive status and occurrence of family separation event) during the active period in south-central Sweden between 2003-2022. The median date for dyadic associations is indicated and day 152 corresponds to June 1.

| Reproductive status | Bear-years | Associations | Average # of associations per female | Median date |
| --- | --- | --- | --- | --- |
| Available | 65 (118) | 5467 | 84 | 146 |
| Cub family | 0 (9) | 0 | 0 | - |
| Cub loss | 13 (18) | 795 | 61 | 165 |
| Yearling family | 7 (16) | 145 | 21 | 201 |
| Yearling separation | 16 (25) | 1600 | 100 | 154 |

**Appendix S6. Difference in association date per group**


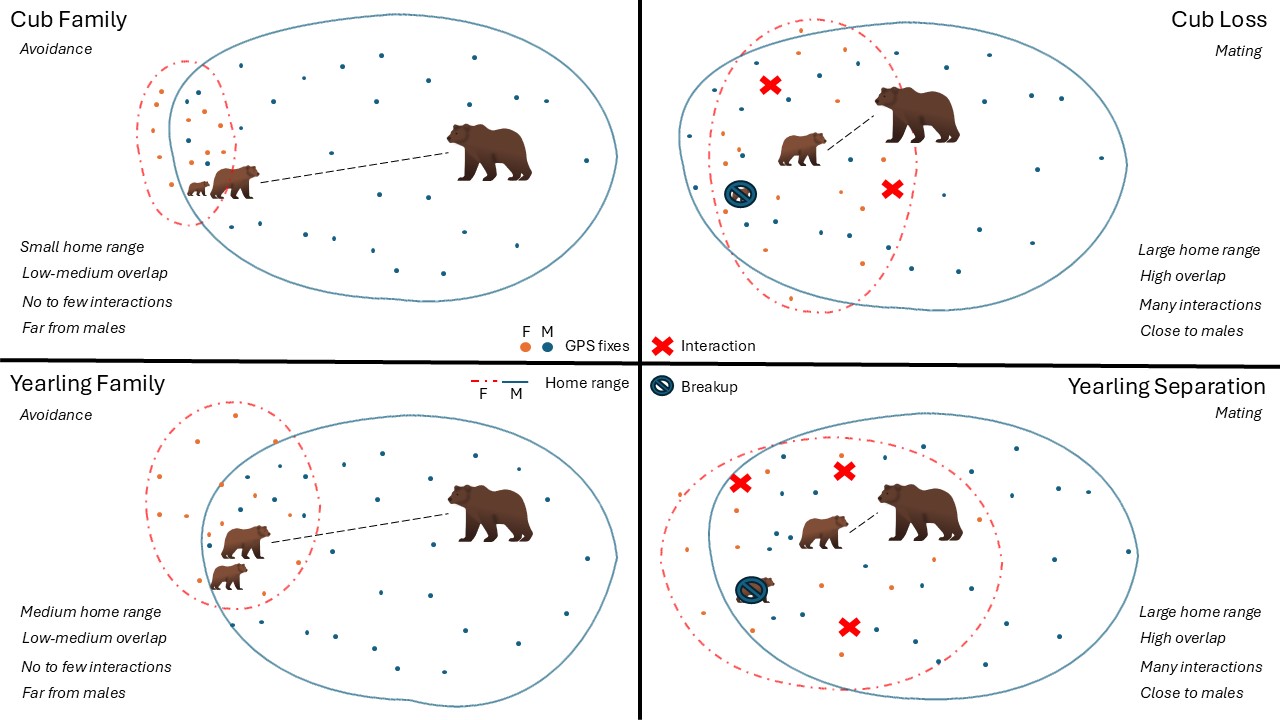


Figure S6: Conceptual figure displaying different aspects of potential behavioral tactics used by brown bears mothers in relation to adult males during the mating season (i.e., “avoidance” or “mating”) based on the age of dependent offspring and the occurrence of family breakup (e.g., offspring loss or separation from offspring). We display four movement and social metrics: 1) female home range size, 2) female-male home range overlap, 3) number of female-male interactions, and 4) proximity of a female to males.

**Appendix S7. Female/family movement behavior throughout active period**

The *amt* package was used to calculate the hourly movement speed and the total number of steps per day (i.e., successful GPS fixes) throughout the active period of all females (excl. hibernation). We determined the total movement distance for all days, between May 1 and October 1, that had at least 20 steps to avoid underestimating daily movement distances.

We fit a hierarchical generalized additive mixed model (HGAM) with a Gamma (log link) distribution to investigate the movement patterns of female classifications. The model included the female classification to identify if there is a significant difference in daily movement distances between the groups. We used the day of the year as a smoother factor. We included an AR1 temporal autocorrelation structure by using the day of the year (i.e., time) combined with the individual ID to take into account any individual based temporal correlation. We also added the unique ID of the female, as a random intercept, to consider individual differences. The model fit was checked using the *mgcViz* package.

We found that females classified as “available”, “Cub loss”, and “Yearling separation” are moving considerable larger daily distances (in km) in comparison to females that remained with the dependent offspring during the mating season (Fig. S7).


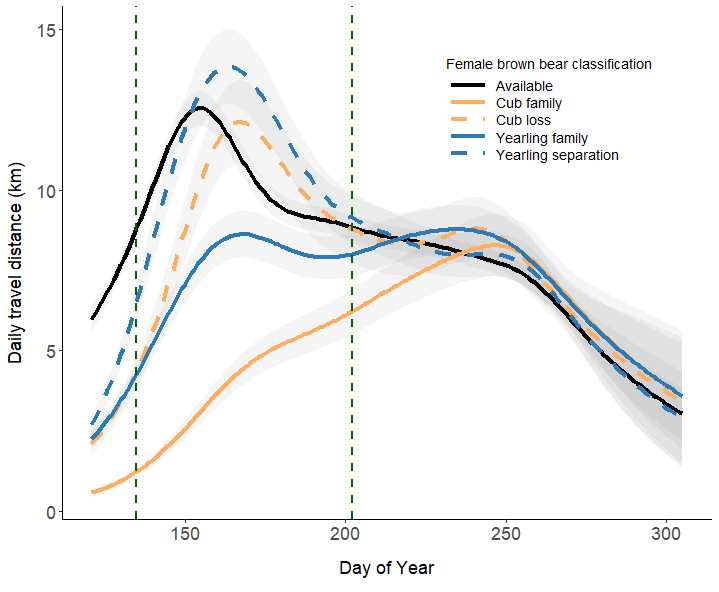


Figure S7: The average daily distance travelled by female brown bears throughout their active period (excluding hibernation) based on their reproductive status (including age of offspring and occurrence of family breakup). The vertical green dotted lines indicate the start and end of the mating season. The data was collected in central-Sweden (2003-2022).

Table S7.1: Summary statistics for the number of female brown bears (bear-years = 256, *n* = 26 819 daily movement distances) per reproductive status and occurrence of separation event (infanticide or family break-up) used to calculate average daily movement distances between 2003-2022 in south-central Sweden.

| Reproductive status | Separation | Bear-years | Samples |
| --- | --- | --- | --- |
| Available | - | 150 | 15 428 |
| Cub | Family | 20 | 1 836 |
|  | Loss | 19 | 2 099 |
| Yearling | Family | 34 | 3 619 |
|  | Separation | 33 | 3 837 |

Table S7.2: Smooth term estimates (log-transformed) from the movement HGAM analysis (reference = available females) regarding the average daily movement distance by female brown bears per reproductive status and occurrence of infanticide or family break-up event per female classification (256 bear-years, *n* = 26 819 daily movement distances).

| **Average daily distance females – males (log-transformed)** | | |
| --- | --- | --- |
| *Fixed coefficients* | *β coefficient* | *p* |
| Available (intercept) | 2.17 | **<0.001** |
| Cub family | -0.70 | **<0.001** |
| Cub SSI | -0.12 | **0.010** |
| Yearling family | -0.21 | **<0.001** |
| Yearling separation | -0.01 | 0.746 |
| *Smooth term^a^* | *edf* | *p* |
| Available * doy | 8.94 | **<0.001** |
| Cub family * doy | 6.30 | **<0.001** |
| Cub SSI * doy | 7.16 | **<0.001** |
| Yearling family * doy | 7.08 | **<0.001** |
| Yearling separation * doy | 7.80 | **<0.001** |

^a^ We used the day of the year (*doy*) variable as a factor-smoother interaction in the GAMM.

**Appendix S8. Difference in association date per group**

See Appendix S5 for information on individuals and samples per group. We used these data to investigate if the association dates of the groups were different with a pairwise non-parametric Wilcoxon rank sum test (Wilcoxon, 1945). We used *p* < 0.05 to identify significant differences between the groups.

We found significant differences between all included groups (Wilcox rank sum test; p < 0.001; Fig. S7), indicating that female-male associations, based on the female classification, are occurring at different times during the year. Males seems to utilize a “staggered” mating tactic, first focusing on solitary females, then females with yearlings, and lastly females with cubs.


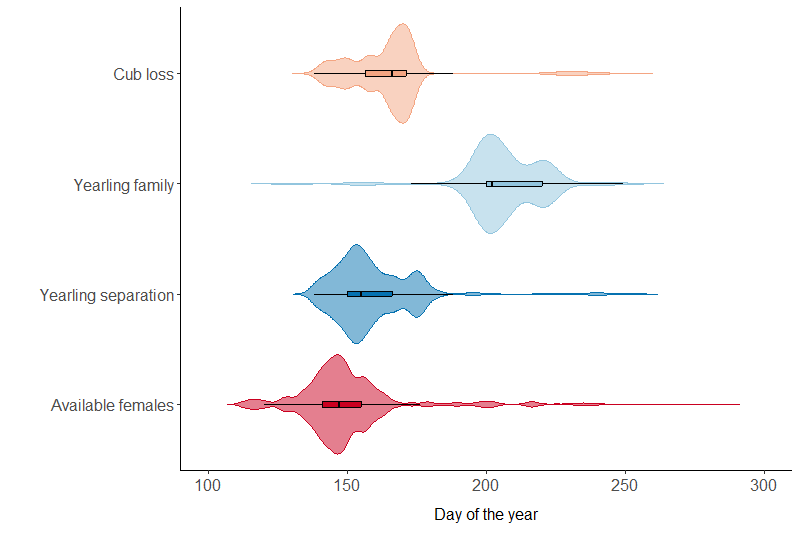


Figure S8: Violin plots, with similar “forced” widths, based on the association dates for brown bear family groups based on offspring age and occurrence of family breakup. The boxplots in the violin plots correspond to the median association date and 5-95% quantiles per group.
